# New York Neurological Society

**Published:** 1884-05

**Authors:** 


### Society Reports.

---

### NEW YORK NEUROLOGICAL SOCIETY.

Stated meeting, March 4, 1884. W. J. Morton, President, in the chair.

First Paper.—Dr. C. L. Dana read a paper upon "Morbid Somnolence," relating a number of histories illustrating different forms of this affection. These forms are classified as follows:

1. Epileptoid sleeping-states.

2. Hysteroid sleeping-states, including (a) spontaneous or "mesmeric" sleep; (b) trance and lethargic states.

3. Morbid somnolence, the expression of a distinct neurosis, (narcolepsy.)

4. Unclassified forms.

The speaker's first case (illustrating class 3) was that of a young man of wealthy family and personal history, who would go to bed at the ordinary hour and could not be roused till noon, or afternoon, or evening of the next day. This would continue for a week or two, when the symptoms would remit.

A second case (illustrating class 3) was that of a young lady who had short attacks of catalepsy, cataleptic *petit-mal*, alternating with sudden attacks of sleep. These came on several times daily. Three other cases, (illustrating class 3) were of neurasthenic persons, who for several months had persistent drowsiness, not attributable to any nutritive or organic disorder.

Dr. Dana also reported a case furnished by Dr. L. Putzel, illustrating the epileptoid sleeping states.

DISCUSSION ON DR. DANA'S PAPER.

Dr. Wm. M. Leszynsky—"I know of two cases which might be termed a mild form of morbid somnolence, where the patient would fall asleep at almost any hour of the day while reading or conversing, the sleep lasting at times for an hour or more.

The cause of this somnolence seemed to me to be undoubtedly due to faulty assimilation of food, and was cured by the use of nitro-muriatic acid, etc.

Dr. Weber—I have seen but a few cases. In diabetes, morbid somnolence is believed to be a prominent symptom. I have seen twenty or thirty of such cases, well pronounced, but have not seen one case where morbid somnolence prevailed; on the contrary, the patients did not sleep as much as normal.

I remember two cases of locomotor ataxia, in which there was a great tendency to prolonged sleep. In one of these cases the man would sleep often fifteen hours at a time.

I have observed sopor in chronic endarteritis in a number of cases, especially in cases where the condition of cerebral arteries tends to apoplexy. There was one man who would fall asleep during dinner, be taken up to bed, and there sleep till the next day.

Drs. Roberts and C. E. Nelson made remarks, giving cases, as to making up sleep-time after prolonged vigil. Dr. Roberts remarked that sopor was met with in his case of myxœdema, read previously before this society, and published. In such cases sopor is recognized as a symptom of disease.

Dr. Shaw, of Brooklyn, related a case of a man who would fall asleep in the clinic.

Dr. R. B. Prescott said: I have one case bearing on this subject, Mr. President, which came into mind while Dr. Dana was reading his paper, and which, as it may not be altogether without interest, I will relate. It is that of a farmer, unmarried, forty years of age or more, living in a small village in Massachusetts, who, some ten years ago, began, without any apparent cause, to be troubled with excessive drowsiness. It manifested itself first in a disposition to sleep unseasonably long in the morning. He would remain in bed until long after the breakfast hour, and complain at intervals during the day of still feeling sleepy. Gradually he came to neglect the work of his farm,

---

This paper will appear in full in the April number of the *Journal of Nervous and Mental Disease*.

and remained about the house, dozing away a considerable portion of the time. His social nature, too, underwent a decided change. He became reserved and silent. He shunned all intercourse with friends and acquaintances, was with difficulty made even to answer ordinary questions, and was easily moved to tears. On one occasion, I was told, that he fell asleep on his wagon while taking a load of produce to the nearest market town, and slept soundly for many hours, his horse having of his own will taken an unfrequented road, and finally stopped at the place where he was discovered, the driver still fast asleep.

His condition at present is that of a gradually deepening mental lethargy. He passes a large portion of his time in bed, and takes little interest in what takes place around him, though at times he partially arouses, and will read the newspapers or carry on a brief conversation, mainly in monosyllabic replies to questions. His bodily functions are all normal, and there is no evidence of any physical disease. His general health was good up to the time of the appearance of this morbid somnolency, and he is not the subject of any hereditary taint, so far as known. He is now regarded by those who know him as mildly insane, and his recovery is not expected.

The President said: I have seen and treated but one of these very peculiar cases which I should be willing, following Dr. Dana's lines of diagnosis, to classify as true morbid somnolence. Of course, those who sleep after prolonged forced wakefulness do not fall within the author's categories.

As an instance of simple sleep of this nature, I well remember of sleeping twenty-four hours, without a moment of recollected consciousness, after two days and two nights in the saddle during a time of great danger. This may be said to be simply normal somnolence. The case of morbid somnolence I refer to, was that of a physician in this city, who had suffered from this condition for fifteen years. He was habitually overcome by an uncontrollable desire to sleep during the day time, no matter how malapropos the time or place; this desire he would fight against with all his power of control, but would finally yield to sopor. Even in the dentist's chair, while a sensitive tooth was being "scraped," he had fallen asleep. Often, in the rounds of daily practice, he would feel this lethargy creeping over him at critical moments, as for instance when his services were most needed at a confinement, and would be forced to yield to it and sleep. It was impossible, for the same reason, for him to read or study. In fact, life was becoming to him a soporific blank.

Other symptoms were forgetfulness, frontal and occipital headache, a general malaise, great sense of weariness, palpitation of the heart on active exercise, and prostatic irritation. He had been examined time and time again by friends of eminence in the medical profession for organic disease, and none existed. The urine, especially, had been the subject of careful tests. I repeated these examinations, with no better results. Malaria was out of the question. I treated this patient on the basis of a profound anæmia—gave him large and increasing doses of iron (Bland's pills), until he was taking 30 grains three times daily; gave him, additionally, glonoin. Under this treatment he improved wonderfully, and at his last visit, several months ago, he reported that he seldom fell asleep during the day.

Dr. Dana, in closing the discussion, reported a case similar to that of the English farmer; this case would have periods of remission for several years. These cases are supposed to end in insanity. There is persistent drowsiness in diabetes and in syphilis, also previous to attacks of epilepsy. There is recognized a "sleeping sickness" in Africa; the French authority, Ballet, mentions these conditions.

Second paper—Treatment of Wry Neck by Sulphate of Atropia, by W. M. Leszynsky, M.D.

The reader related the history of the case of a young woman, whose occupation being that of a book-folder, she was obliged to turn her head very frequently toward the left side. The right sterno-cluilo-mastoid and trapezius muscles became affected with a very severe form of clonic spasm, which almost exhausted the strength of the patient. The treatment adopted was the daily injection of sulphate of atropia into the contracting muscles, beginning with gr. 1.80, and gradually increasing to gr. 1.6, which maximum dose was continued four days, when recovery supervened.

In addition to the atropia, galvanism was used, and the faradic current was applied to the opposite side.

DISCUSSION.

Dr. J. C. Shaw—I have been called three times in consultation in these cases where atropine was used. There was a great deal of pain, and marked neuropathic tendency; insanity in the family, in one case. There is one difficulty in the treatment by atropine, that it sometimes causes disagreeable symptoms, especially in delicate women. In one case, where the drug was pushed, it caused such distress that the patient, a woman, refused to take it longer. Atropine in large doses cannot be used in all cases, therefore.

Dr. C. L. Dana said that Dr. Leszynsky was entitled to great credit, in employing atropia against such physiological odds. He believed that the cure was due to the employment of atropia. One point must be borne in mind, and that is that we must select our cases; in those cases where the disease is plainly neurosis, atropia may answer. In many cases, however, the disease appears to be of a peripheral and rheumatic character. Here antirheumatic remedies answer better.

Dr. Gibney—In view of the fact that Dr. Leszynsky administered electricity and other agents, as his report shows, some doubt might be expressed as to the curative effects of the atropine injections. The relationship of cause and effect does not seem sharply enough defined. I have had no personal experience with this drug in torticollis. A few years ago, in a case of rotary spasm of the head, I had very prompt and excellent results in the use of the fluid extract of gelseminum carried to tonic doses. Dr. Leszynsky certainly deserves credit for the heroic dosage of atropine in this case.

Dr. Birdsall related the history of a case of torticollis treated at the Manhattan Hospital by his assistant, Dr. Terriberry, in a child about 8 years of age, by the application of as strong a galvanic current as could be endured for from twenty to thirty minutes on the affected muscles, three times a week for several weeks, with gradual improvement, which finally terminated in complete recovery. Ticture of belladonna was administered in drop doses, until slight physiological effects were produced. Dr. Birdsall was inclined to credit the curative effect in this case mainly to the galvanism, though he thought that a combination of the method with atropia and that of galvanism would, in general, be far more serviceable than either alone.

Dr. Weber—Was a traumatic effect produced by the hypodermic injections?

Dr. Leszynsky—The injections were made into the substance of the muscle, and no traumatic effect was produced. The preparation of atropia used was Merck's, and the solution was freshly prepared every two or three days.

Remarks of Dr. David Webster:

Mr. President:—I have listened to Dr. Leszynsky's paper with much interest. Although I have seen but few cases of wry neck, I have had a good deal of experience with atropine, and I beg leave to question whether the same results might not have been accomplished by smaller doses applied locally. For the purpose of relaxing the sphincter pupillæ and the ciliary muscle we never give atropia by the mouth or hypodermically, but always apply it locally to the surface of the eyeball. Less than 1-20000 of a grain applied to the conjunctiva will paralyze the muscles I have named, while it would require a many times larger dose to produce the same effect, if given hypodermically.

It is remarkable that Dr. Leszynsky's patient tolerated so large a dose as one-sixth of a grain. There is a wide difference in the quantity required to produce the physiological effects of the drug in different persons. I have frequently seen a drop of a four-grain solution, applied to the eye, produce the peculiar scarlet flushing of the face, especially in infants. I also know of a case in which a single drop in the eye caused marked delirium in a young lady, so that she had to be taken home in a carriage. I have had some personal experience with the physiological effects of atropia. I once swallowed what I supposed to be ten drops of Magendie's solution of morphia, to check a diarrhœa, while I went to Brooklyn to assist in an enucleation. On the way I noticed that I felt very strangely, going off into curious dreams, entering into imaginary conversations, etc. When I got to the place of operation, I found on attempting to talk that I

could scarcely speak above a whisper, my mouth and throat were so dry. Dr. Agnew noticed that my face was flushed and my pupils dilated. I went home and went to bed, and slept soundly until the next morning. As soon as I awoke, it dawned upon me that I must have taken atropine instead of morphine. As soon as I saw Dr. Agnew, he told me he had arrived at the same conclusion. I found the atropine and morphine bottles side by side on my table. The mystery was explained.

I once saw a case in the practice of a brother practitioner, where one-sixteenth of a grain of sulphate of atropia, given with half a grain of morphia subcutaneously, produced delirium, lasting for half a day or more. This was in a hysterical lady who was used to hypodermics of morphia without atropia.

Dr. Leszynsky's method of giving the drug was a perfectly safe one, however, as he cautiously felt his way, from smaller to larger doses.

Dr. G. W. Jacoby said: It was not my intention to make any remarks upon this subject, as the objection which I intended to raise to the indiscriminate employment of galvanism and atropine in the treatment of Dr. L's case, has already been made by some of the preceding speakers; but Dr. Gibney's remarks in reference to the facility of producing the physiological effects of atropine, in some cases, by very minute doses, recall to my mind very vividly a case in which this was also very noticeable. The patient, a girl, aged 12 years, came to me affected with a left-sided tonic torticollis, probably of rheumatic origin. My results with electricity upon other cases having been unsatisfactory, I determined to treat this case by the hypodermic injection of sulphate of atropia. I therefore injected one-fiftieth of a grain of the drug.

This one injection produced all the symptoms of atropine-poisoning, ending in a violent delirium which lasted for ten hours.

When the patient had recovered from the effects of the atropine, I naturally felt reluctant to continue its use, and began treatment of the torticollis by galvanism. After two weeks the child was discharged from treatment entirely recovered.

The points that I wish to mark, are first, the small amount of atropine necessary in this case to produce delirium, and secondly, the fact of a cure by self-limitation, or possibly through the action of the galvanic current. Had no ill effects resulted from the use of the atropia, I would probably have continued its use, and my patient recovering, it would have been only natural to attribute this recovery to the use of the atropine.

Therefore, we cannot be too cautious in drawing conclusions from a single case, no matter how well observed, and we should be very careful not to use two potent remedies, such as galvanism and atropine, simultaneously, as our scepticism in regard to the efficiency of either one will not be considered scientific proof of the beneficial action of the other.

Dr. Leszynsky, in closing the discussion, said:

As Dr. Dana saw the patient referred to in my paper, I am pleased to hear that he agrees with me in stating that recovery was due to the employment of the atropia.

In reporting the history of this case, I expected that the question would arise as to which of the remedies employed had effected the cure, therefore I was not surprised to hear the criticism of Drs. Gibney and Jacoby, and in reply I will state that the number of cells used in applying the galvanic current was from ten to twenty of a Stohrer portable battery. The patient could not tolerate a stronger application, and this was continued for nearly fifteen minutes daily. After the removal of the electrodes, I found that the spasm invariably became more vigorous than ever, and I always allowed about ten minutes to elapse before injecting the atropia.

I would again direct the attention of the society to the fact that, notwithstanding the daily application of galvanism in conjunction with the use of atropia, *no improvement was shown until the twentieth day, soon after a rapid increase of the atropia from gr. one-twentieth to nearly gr. one-eighth.* Then the improvement became so evident that it can hardly be doubted that the atropia was the important element which effected the successful result. In regard to the use of the bromide of sodium, I can safely say that bromism was not produced. The faucial reflex was frequently tested, and remained well marked throughout the entire course of treatment.

Dr. Webster's suggestion may be a very good one if we accept it from an ophthalmological standpoint, but in this class of cases I cannot see what advantage could be gained by the inunction of the oleate of atropia.

The object in using this sulphate of atropia was to produce paralysis of the trunk and branches of the spinal accessory nerve, therefore it was injected into the substance of the muscle for the purpose of producing its *local effects* on the motor nerve, although eminent authorities like Ringer and Traser have concluded, after an elaborate series of experiments upon living animals, that atropia paralyzes the motor nerves through its action upon the spinal cord, and not by its action through the circulation. I believe that the oleate, if applied locally, would produce more rapid constitutional symptoms on account of its speedy absorption, and another objection is that the dose cannot be so accurately determined.

In conclusion, I will state that the patient remains well, and that no sign nor symptom of spasm has since been shown.

Nomination of officers for ensuing year: President, Birdsall, Gray, Morton, W. A. Hammond; First Vice-President, C. L. Dana; Second Vice-President, G. W. Jacoby; Recording Secretary, E. C. Wendt; Corresponding Secretary, W. M. Leszynsky; Treasurer, E. C. Harwood; Councillors, (five) Weber, Seguin, Jacoby, Morton, W. A. Hammond, McBride.

The society then adjourned.

